# The NONEXPRESSOR OF PATHOGENESIS-RELATED GENES 1 (NPR1) and Related Family: Mechanistic Insights in Plant Disease Resistance

**DOI:** 10.3389/fpls.2019.00102

**Published:** 2019-02-13

**Authors:** Robert Backer, Sanushka Naidoo, Noëlani van den Berg

**Affiliations:** ^1^Forestry and Agricultural Biotechnology Institute, University of Pretoria, Pretoria, South Africa; ^2^Department of Biochemistry, Genetics and Microbiology, University of Pretoria, Pretoria, South Africa

**Keywords:** NPR1, NPR1-like, systemic acquired resistance, salicylic acid, plant disease, *pathogenesis-related*

## Abstract

The NONEXPRESSOR OF PATHOGENESIS-RELATED GENES 1 (NPR1) and related NPR1-like proteins are a functionally similar, yet surprisingly diverse family of transcription co-factors. Initially, NPR1 in *Arabidopsis* was identified as a positive regulator of systemic acquired resistance (SAR), paralogs NPR3 and NPR4 were later shown to be negative SAR regulators. The mechanisms involved have been the subject of extensive research and debate over the years, during which time a lot has been uncovered. The known roles of this protein family have extended to include influences over a broad range of systems including circadian rhythm, endoplasmic reticulum (ER) resident proteins and the development of lateral organs. Recently, important advances have been made in understanding the regulatory relationship between members of the NPR1-like protein family, providing new insight regarding their interactions, both with each other and other defense-related proteins. Most importantly the influence of salicylic acid (SA) on these interactions has become clearer with NPR1, NPR3, and NPR4 being considered *bone fide* SA receptors. Additionally, post-translational modification of NPR1 has garnered attention during the past years, adding to the growing regulatory complexity of this protein. Furthermore, growing interest in *NPR1* overexpressing crops has provided new insights regarding the role of NPR1 in both biotic and abiotic stresses in several plant species. Given the wealth of information, this review aims to highlight and consolidate the most relevant and influential research in the field to date. In so doing, we attempt to provide insight into the mechanisms and interactions which underly the roles of the NPR1-like proteins in plant disease responses.

## Introduction

The *NONEXPRESSOR OF PATHOGENESIS-RELATED GENES* 1 (*NPR1*), as well as *PATHOGENESIS-RELATED* (*PR*) genes, play a fundamental role in a plant’s response to pathogen challenge. NPR1 plays a significant role in the establishment of systemic acquired resistance (SAR) as well as induced systemic resistance (ISR) (Pieterse et al., 1998); it acts as the master key to the plant defense signaling network, mediating cross-talk between the salicylic acid (SA) and jasmonic acid/ethylene (JA/ET) responses. Constitutive *NPR1* expression within wild-type *Arabidopsis thaliana* ensures a quick response to SA (Cao et al., 1998). NPR1 is then translocated primarily to the nucleus where it indirectly activates *PR* gene expression by recruiting TGA transcription factors (Zhang et al., 1999; Despres et al., 2000; Zhou et al., 2000; Kim and Delaney, 2002). The exact mechanisms involved in NPR1 activation, as well as NPR1-dependent/independent *pathogenesis-related* (*PR*) gene expression and the overall role of NPR1 in pathogen defense are important topics of study.

The mechanism by which SA activates NPR1 is not completely understood, yet a lot has been uncovered in recent years. During non-stress conditions, NPR1 can be found as a large cytoplasmic oligomer (Mou et al., 2003). An oxidative burst observed during SA-induced SAR results in a reducing environment as the cell recovers (Mou et al., 2003). This redox state then contributes toward NPR1 monomerization, nuclear localization and *PR* gene expression. Activation of various TGA transcription factors occurs under these conditions (Despres et al., 2000). SA is believed to achieve reducing conditions in two stages: (1) induction of oxidative stress reducing genes (2–3 h after SA treatment) and, (2) NPR1 dependent *PR* gene expression (12–16 h after SA treatment) (Horvath and Chua, 1996; Dong, 2004; Uquillas et al., 2004). Expression of *PR* genes are essential for the development of SAR, *Arabidopsis* mutants deficient in NPR1 show reduced *PR* gene expression and increased susceptibility to pathogens (Cao et al., 1994; Roetschi et al., 2001). Hence NPR1 plays an integral part in the efficacy of plant immune responses.

In studied plant species, two to six *NPR1*-like genes have been found. This family of proteins contain ankyrin repeats and Broad Complex, Tramtrack and Bric a brac/Pox virus and Zinc finger (BTB/POZ) domains, two well documented protein–protein interaction domains (Bardwell and Treisman, 1994; Cao et al., 1997; Ryals et al., 1997; Aravind and Koonin, 1999; Hepworth et al., 2005; Li et al., 2006; Spoel et al., 2009). Phylogenetic analysis separates the NPR1-like proteins into three distinct clades, suggesting functional divergence (Hepworth et al., 2005; Zhang et al., 2006). Yet, significant overlap in clade function can be found within the first two clades involved in positive and/or negative SAR regulation (Liu et al., 2005; Le Henanff et al., 2009). The third clade seems to be involved in the development of growing tissues (Hepworth et al., 2005). The varied functional role of NPR1-like proteins suggests a complex functionally important family involved in plant immune responses and development. Thus, this review aims to examine and consolidate these functions, providing mechanistic insights regarding pathogen response.

## Overview of Plants Responses to Pathogens

The interaction between plants and their pathogens has been studied in some detail. Plant–pathogen interactions involve a wide variety of systems on both sides, the balance of which determines the success of either the host or the pathogen (Lodha and Basak, 2012). Compatible interactions occur when a plant is unable to coordinate effective defense responses, enabling the pathogen to colonize and proliferate within the host (Schenk et al., 2000). In contrast, an incompatible interaction occurs when defense responses are sufficient at preventing spread of the pathogen within host tissues (Hammond-Kosack and Jones, 2000). Successful plant immunity relies on both non-specific preformed and inducible defense mechanisms as well as specific induced immune responses. The first line of defense includes physical barriers such as waxy layers, rigid cell walls, antimicrobial compounds and secondary metabolites (Agrios, 2005; Reina-Pinto and Yephremov, 2009). Microbes which overcome these preformed defenses trigger the next line of immune responses. The first of these, pattern-triggered immunity (PTI) is induced by the action of pathogen-recognition receptors (PRRs) which recognize microbe-associated molecular patterns (MAMPs) preventing further invasion of host tissues (Jones and Dangl, 2006; Zipfel, 2009). Additionally, a subset of molecules referred to as damage-associated molecular patterns (DAMPs), are passively released from damaged native plant tissue and capable of activating and perpetuating innate immune responses (Choi and Klessig, 2016).

With the aim of overcoming PTI, pathogens secrete effector molecules which target specific host proteins, manipulating host processes with the purpose of enhancing virulence, a state referred to as effector triggered susceptibility (ETS) (Jones and Dangl, 2006). In response, intracellular resistance (R) proteins, most of which are nucleotide-binding site leucine-rich repeat (NBS-LRR) proteins, monitor the status of effector targeted plant proteins or bind directly to them, initiating defense responses in case of attack (Van der Biezen and Jones, 1998; Jia et al., 2000; Dangl and Jones, 2001; Deslandes et al., 2003). These processes initiate effector triggered immunity (ETI), most often characterized by rapid localized programmed cell death (PCD) also known as the hyper-sensitive response (HR), which prevents further spread of the pathogen (Goodman and Novacky, 1994; Van Loon, 1997; Jones and Dangl, 2006). Tissues distal to the initial site of infection experience an increased accumulation of several defense signals, including SA (An and Mou, 2011; Fu and Dong, 2013). Subsequently, systemic production of a collection of pathogen-induced antimicrobial proteins known as PR proteins increase, which enhance resistance to a variety of pathogens (Van Loon and Van Strien, 1999; Durrant and Dong, 2004). This signifies the establishment of SAR, a long lasting systemic broad spectrum resistance which is effective at preventing infection by a wide variety of pathogenic bacteria, fungi, oomycetes, viruses and nematodes (Ryals et al., 1996; Sticher et al., 1997). These basic defenses are intricately interwoven with numerous interactions both within and amongst pathways, which through coordinated signaling events comprise plant immunity.

## Salicylic Acid and Phytohormone Cross-Talk

Salicylic acid is a phenolic compound produced by various prokaryotes and eukaryotes (An and Mou, 2011). In plants, its role is as a phytohormone essential to PTI, ETI, and SAR induction (Pieterse et al., 1998; Durrant and Dong, 2004; Tsuda et al., 2009). Whereas the JA/ET signaling pathway is essential for defense against herbivores, insects, and necrotrophic pathogens, the SA signaling pathway is crucial to immune responses against biotrophic and hemibiotrophic pathogens (Shah, 2003; Howe and Jander, 2008). Such pathogen challenge induces the production of endogenous SA which is vital in establishing SAR (Malamy et al., 1990; Metraux et al., 1991; Rasmussen et al., 1991; Gaffney et al., 1993; Delaney et al., 1994). Consequently, mutants deficient in the accumulation of SA such as *SA induction-deficient 2* (*sid2*) or *enhanced disease-susceptibility 5* (*eds5*) and plants expressing the salicylate hydrolase *nahG* gene, display compromised SAR induction (Nawrath and Métraux, 1999; Wildermuth et al., 2001; Nawrath et al., 2002; van Wees and Glazebrook, 2003). Thus, an inability to synthesize or accumulate SA is directly correlated to increased susceptibility to certain pathogens (van Wees and Glazebrook, 2003). Interestingly, SA influences various other hormone signaling pathways including JA and ET as well as auxin (Vlot et al., 2009). In general the balance between these hormones governs the bulk of host defense signaling (Robert-Seilaniantz et al., 2011). This is evident through heightened biotroph resistance resulting in increased susceptibility to necrotrophs and vice versa (Robert-Seilaniantz et al., 2011).

The biosynthesis of SA relies on two pathways, (1) the cinnamic acid pathway which requires PHENYLALANINE AMMONIA LYASE (PAL) and (2) the isochorismate pathway requiring ISOCHORISMATE SYNTHASE (ICS) and ISOCHORISMATE PYRUVATE LYASE (IPL) (Verberne et al., 2000; Wildermuth et al., 2001; Strawn et al., 2007; Chen et al., 2009b; Vlot et al., 2009). The isochorismate pathway is regarded as the predominant biosynthetic pathway during pathogenic threat, evinced by *Arabidopsis ics* mutants which accumulate significantly lower levels of SA following pathogenic stress (Wildermuth et al., 2001; Garcion et al., 2008), a statement also true in *Nicotiana benthamiana* and *Solanum lycopersicum* (Uppalapati et al., 2007; Catinot et al., 2008). Several derivatives of SA exist *in planta* such as SA *O*-β-glucoside (SAG), salicyloyl glucose ester (SGE), methyl salicylate (MeSA), methyl salicylate *O*-β-glucoside (MeSAG) and 2,5 dihydroxybenzoic acid (gentisic acid) (Shulaev et al., 1997; Lee and Raskin, 1998; Seskar et al., 1998; Belles et al., 1999; Song, 2006; Park et al., 2007; Dean and Delaney, 2008). Notably gentisic acid is essential to activating a specific set of *PR* genes (Belles et al., 1999). In fact, many of the aforementioned derivates perform specialized roles in plant immune responses and are required for the complete induction of SA-dependent defense responses, although some are still subject to debate (Nobuta et al., 2007; Vlot et al., 2009; Zheng et al., 2012; Fu and Dong, 2013). Most notably, MeSA has been proven to act as a signal for SAR in tobacco, *Arabidopsis* and potato (Park et al., 2007; Vlot et al., 2008). However, in *Arabidopsis* extended exposure to light following infection can negate the need for MeSA to signal systemic SAR development (Liu et al., 2011). In addition, MeSA might also serve as a volatile cautioning signal to neighboring plants (Koo et al., 2007; Spoel and Dong, 2012). Other compounds such as SAG and SGE ensure an ample supply of SA during pathogen challenge as bioactive free SA is readily hydrolyzed from inactive SAG stored within the vacuole (Dean et al., 2005).

The role of SA in disease resistance is certainly significant in all plant species (Malamy et al., 1990; Gaffney et al., 1993; Delaney et al., 1994; Vernooij et al., 1994; Lawton et al., 1995). Some pathogens even manipulate SA homeostasis to promote host invasion (Feys et al., 1994; Zheng et al., 2012). Several *Pseudomonas syringae* pathovars produce the phytotoxin coronatine, which indirectly represses *ICS1* and activates *BENZOIC ACID/SALICYLIC ACID CARBOXYL METHYLTRANSFERASE 1* (*BSMT1*) expression, which converts SA into MeSA, to suppress SA accumulation (Feys et al., 1994; Zheng et al., 2012). Although not directly responsible for all signal transduction, SA forms an integral part of a complex network responsible for signal transduction. Initially, global transcriptional profiling discovered extensive crosstalk between SA-, JA-, and ET- pathways in *Arabidopsis* (Glazebrook et al., 2003). Microarray expression profiling in *Arabidopsis* also demonstrated the true extent of cross-talk between various defense signaling pathways (Schenk et al., 2003). This interconnected signaling network serves to fine-tune defense responses through both antagonistic and synergistic interactions (Salzman et al., 2005).

Crosstalk between signaling networks is essential to spatial, temporal and plant–pathogen interaction specificity which informs trade-offs during challenge by multiple biotic and abiotic stresses (Spoel and Dong, 2008). However, phytohormone crosstalk can often be manipulated by pathogens to increase virulence (Spoel and Dong, 2008). Coronatine produced by virulent *P. syringae* for instance, which structurally mimics jasmonyl-L-isoleucine (JA-Ile), stimulates JA responsive pathways thereby suppressing SA signaling (Feys et al., 1994; Bender et al., 1999; Koornneef and Pieterse, 2008). Furthermore, coronatine accumulation is associated with increased abscisic acid (ABA) biosynthesis which in turn leads to reduced SA accumulation, basal defense gene expression and ultimately heightened susceptibility (de Torres-Zabala et al., 2007; Mohr and Cahill, 2007). It has also been clearly demonstrated that auxin, a primary growth hormone, antagonizes SA accumulation (Wang et al., 2007). To this effect, the *P. syringae* effector AvrRpt2 manipulates auxin homeostasis to promote virulence (Chen et al., 2007). These examples highlight the extent of crosstalk between phytohormone pathways which extends beyond SA and JA/ET antagonism, providing insight into the mechanisms plants use to overcome pathogenic threat (Spoel and Dong, 2008; Robert-Seilaniantz et al., 2011). For instance, in wild-type *Arabidopsis* infected with *P. syringae* the accumulation of SA represses the JA mimicking effects of coronatine, while SA indirectly prevents the degradation of the auxin repressor AXR2 thereby avoiding the expression of auxin-responsive genes (Spoel et al., 2003; Wang et al., 2007). Thus it is clear that the fundamental link in phytohormone crosstalk is the regulation of SA synthesis (Fu and Dong, 2013). For an in-depth review of the topic see (Robert-Seilaniantz et al., 2011; Berens et al., 2017).

## Nonexpressor of Pathogenesis-Related Genes 1

An indispensable player in the SA-defense response pathway is NPR1, a protein involved in fundamental responses to pathogenic challenge. The search for a SA responsive protein led to the discovery of NPR1, a positive regulator of SAR (Glazebrook et al., 1996; Cao et al., 1997; Ryals et al., 1997; Shah et al., 1997). *Arabidopsis npr1* mutants display increased disease susceptibility and a decrease in SAR-triggered *PR* gene expression, specifically *PR1* and *PR5* (Cao et al., 1994; Glazebrook et al., 1996). Whereas complementing *npr1* mutants with wild-type *NPR1* restores resistance and *PR* gene expression (Cao et al., 1997). Various plant species overexpressing *AtNPR1* or its orthologs display enhanced disease resistance to a wide range of pathogens (Cao et al., 1998; Chern et al., 2001, 2005b; Lin et al., 2004; Makandar et al., 2006; Xujing et al., 2006; Malnoy et al., 2007; Potlakayala et al., 2007; Yuan et al., 2007; Wally et al., 2009; Parkhi et al., 2010b; Le Henanff et al., 2011; Kumar et al., 2013; Dutt et al., 2015; Molla et al., 2016). Furthermore, compelling evidence identifies NPR1 as a key element in the crosstalk between the SA and JA/ET responses (Spoel et al., 2003). Hence NPR1 plays a significant role in a broad range of defense responses, acting as the master regulator of plant defense signaling (Dong, 2004).

Specialized domains, specifically the ankyrin repeat and the BTB/POZ domains, facilitate protein–protein interactions (Bardwell and Treisman, 1994; Cao et al., 1997; Ryals et al., 1997; Aravind and Koonin, 1999; Hepworth et al., 2005; Li et al., 2006; Spoel et al., 2009). While a bipartite nuclear localization sequence allows for nuclear localization of NPR1 following SA induction (Kinkema et al., 2000). Consequently a subset of the TGA family of basic domain/leucine zipper (bZIP) transcription factors are activated by NPR1 leading to expression of *PR* genes and SAR induction (Zhang et al., 1999; Kinkema et al., 2000; Zhou et al., 2000; Despres et al., 2003). These data suggest that NPR1 is a transcription cofactor responsible for effecting SA-dependent signaling, a concept supported by genome-wide expression analysis of *npr1* mutants (Wang et al., 2006). However, recent advances provide a clearer, malleable and intricate picture of NPR1-dependent defense responses.

## NPR1-Like Family

Numerous NPR1-like proteins, both putative and confirmed, have been identified in various plant species (Table 1). All described NPR1-like proteins contain an ankyrin repeat domain and BTB/POZ domain indicating high levels of functional conservation in the NPR1-like family (Bardwell and Treisman, 1994; Cao et al., 1997; Ryals et al., 1997; Aravind and Koonin, 1999; Hepworth et al., 2005; Li et al., 2006; Spoel et al., 2009). However, as the list of NPR1-like proteins in different plant species increases, so does the complexity and variability in function. In *Arabidopsis* alone, five additional *NPR1*-like genes have been described: *AtNPR2, AtNPR3, AtNPR4, AtNPR5/AtBOP1* and *AtNPR6/AtBOP2* (Ha et al., 2004; Hepworth et al., 2005; Liu et al., 2005; Zhang et al., 2006).

**Table 1:** NPR1-like proteins.

Common name	Latin name	Reference
Arabidopsis	*Arabidopsis thaliana*	Cao et al., 1997; Hepworth et al., 2005; Liu et al., 2005
Apple	*Malus pumila*	Malnoy et al., 2007
Apple	*Malus hupehensis*	Zhang et al., 2012
Rice	*Oryza sativa*	Goff et al., 2002
Poplar	*Populus trichocarpa*	Tuskan et al., 2006
Tobacco	*Nicotiana tabacum*	Liu et al., 2002
Tobacco	*Nicotiana glutinosa*	Zhang et al., 2010c
Grapevine	*Vitis vinifera*	Le Henanff et al., 2009
Norton grapevine	*Vitis aestivalis* cv. *Norton*	Zhang et al., 2013
Cotton	*Gossypium hirsutum*	Zhang et al., 2008
Asian pear	*Pyrus pyrifolia*	Faize et al., 2009
Sweet potato	*Ipomoea batatas*	Chen et al., 2009a
Papaya	*Carica papaya*	Zhu et al., 2003; Peraza-Echeverria et al., 2012
Banana	*Musa acuminata*	Endah et al., 2008
Banana	*Musa* spp. ABB	Zhao et al., 2009
Tomato	*Solanum lycopersicum*	The Tomato Genome, 2012
Mustard greens	*Brassica juncea*	Meur et al., 2006
Soybean	*Glycine max*	Sandhu et al., 2009
Cacao tree	*Theobroma cacao*	Shi et al., 2010
Sugar cane	*Saccharum spp.*	Chen et al., 2012a
Coffee	*Coffea arabica*	Barsalobres Cavallari et al., 2013
Orchid	*Phalaenopsis aphrodite*	Chen et al., 2013
Wheat	*Triticum aestivum* L.	Diethelm et al., 2014
Beet	*Beta vulgaris*	Kuykendall et al., 2007
Avocado	*Persea americana*	Backer et al., 2015
Coconut palm	*Cocos nucifera* L.	Nic-Matos et al., 2017
Gladiolus	*Gladiolus hybridus*	Zhong et al., 2015
Canola	*Brassica napus*	Potlakayala et al., 2007
Peanut	*Arachis hypogaea*	Wu et al., 2014
Oriental lily	*Lilium ‘Sorbonne’*	Wang et al., 2017
Eucalyptus	*Eucalyptus grandis*	Naidoo et al., 2013


*List of plant species which either code for putative or confirmed NPR1-like proteins.*

**FIGURE 1:**
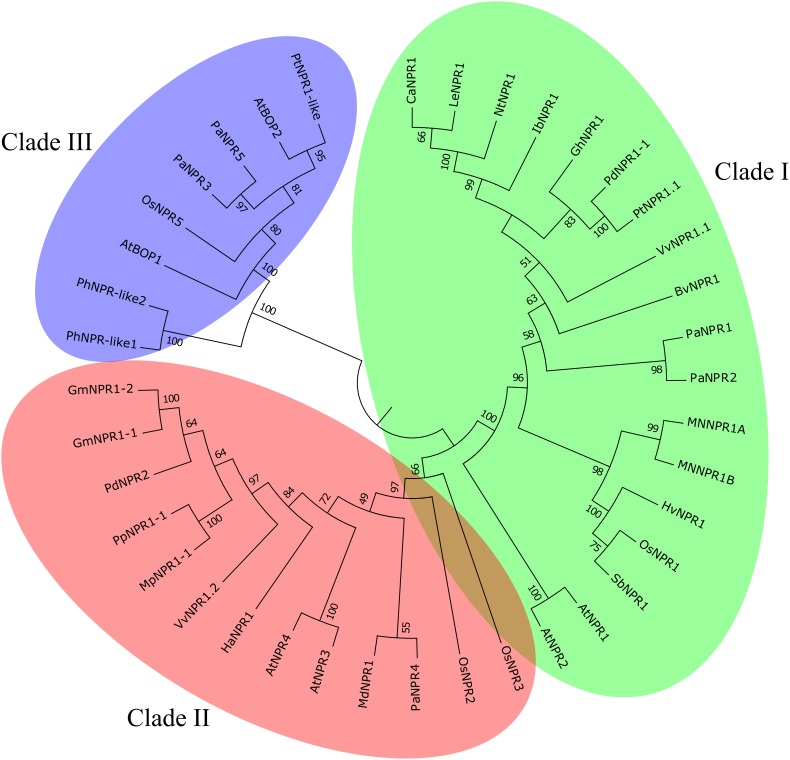
NPR1-like protein family. Three clades of the NPR1-like family of proteins from both vascular and non-vascular plant species. Clade I (AtNPR1 and AtNPR2) contains known positive regulators of SAR while clade II (AtNPR3 and AtNPR4) contains known negative regulators of SAR and clade III contains NPR1-like proteins involved in the development of lateral organs. Adapted from Backer et al. (2015).

Phylogenetic analysis reveals that the NPR1-like family classifies into three clades (Figure 1) (Hepworth et al., 2005; Zhang et al., 2006). Each clade seems to fall into a distinct functional niche. Clade 1 (AtNPR1 and AtNPR2) is involved with positive SAR regulation, clade 2 (AtNPR3 and AtNPR4) with negative SAR regulation and clade 3 (AtBOP1 and AtBOP2) with growth and development of leaves and flowers (Cao et al., 1998; Hepworth et al., 2005; Zhang et al., 2006). These clades are not always functionally robust and as such phylogenetic analyses alone are insufficient for functional annotation (Liu et al., 2005; Zhang et al., 2006; Le Henanff et al., 2009). Nonetheless phylogenetic grouping provides a foundation for understanding functional variability among NPR1-like proteins.

## NPR1 in Crosstalk

Effective defense responses rely on correct activation of either the SA- and JA-defense response pathways (Glazebrook, 2005). Although known to interact synergistically, the SA- and JA-defense response pathways are commonly regarded as antagonistic (Felton and Korth, 2000; van Wees et al., 2000; Glazebrook, 2005; El Oirdi et al., 2011). In so doing, plants ensure minimal fitness loss whilst safeguarding disease resistance (Mur et al., 2006; Koornneef and Pieterse, 2008). Research by Spoel et al. (2003) clearly demonstrates antagonistic crosstalk between the SA and JA pathways. In wild-type *Arabidopsis* the combined exogenous application of SA and MeJA favors activation of the SA-defense response pathway, evident through increased *PR1* expression and simultaneous suppression of JA-responsive defense gene expression (Spoel et al., 2003). Furthermore, the simultaneous infection of *Arabidopsis* with both biotrophic and necrotrophic pathogens results in an increased susceptibility to the latter, indicating JA defense suppression via the SA pathway (Spoel et al., 2003, 2007). However, crosstalk seems to be limited to local tissues, thereby preventing necrotrophic pathogens from capitalizing on the suppressed JA response pathway in systemic tissue (Spoel et al., 2007). Suppression also appears to be mediated by cytoplasmic NPR1 which upon SA induction limits JA dependent signaling (Spoel et al., 2003; Ndamukong et al., 2007; Yuan et al., 2007).

Interestingly some pathogens can manipulate this cross talk to promote disease. A necrotrophic fungus, *Botrytis cinerea*, expresses an exopolysaccharide which induces the SA pathway thereby preventing JA dependent gene expression (El Oirdi et al., 2011). El Oirdi et al. (2011) demonstrated the role of NPR1 in *B. cinerea* pathogenesis by infecting tomato plants in which NPR1 had been silenced. Such NPR1 deficient lines showed significantly reduced disease symptoms. Additionally, transgenic *Arabidopsis* overexpressing NPR1 show enhanced *B. cinerea* susceptibility (El Oirdi et al., 2011). This concept is supported by the observation that NPR1 may play a role in preventing the accumulation of SA during herbivory (Rayapuram and Baldwin, 2007). In NPR1-silenced *Nicotiana attenuata*, SA accumulation was accompanied by an increased susceptibility to herbivores, suggesting that NPR1 might suppress SA production allowing JA-mediated defense responses to dominate (Rayapuram and Baldwin, 2007). Indeed, NPR1 has been shown to prevent the accumulation of SA by negatively regulating *ICS1* upon entry into the nucleus (Zhang et al., 2010a). Additionally, *Arabidopsis npr1* mutants are deficient in mounting ISR (Pieterse et al., 1998). The Cauliflower mosaic virus (CaMV) protein P6 is associated with repressing the SA- and enhancing the JA-defense response pathways (Love et al., 2012). Interestingly, P6 is implicated in the accumulation of inactive NPR1 within the nucleus, an avenue which likely enforces its’ effect (Love et al., 2012). Thus, NPR1 is essential in both the SA and JA/ET pathways, regulating the accumulation of SA to activate the appropriate defense signal.

## Monomerization of NPR1

Although NPR1 is involved in hormone cross talk, its’ main purpose is establishing SAR through *PR* gene expression (Cao et al., 1994; Delaney et al., 1995; Glazebrook et al., 1996; Shah et al., 1997). Achieving this requires the nuclear localization of monomeric NPR1 which interacts with TGA transcription factors to form a transcriptional complex (Zhang et al., 1999; Despres et al., 2000; Zhou et al., 2000; Kim and Delaney, 2002). This complex then associates with an *activation sequence-1* (*as-1*)-like motif within the *PR* promoter (Lebel et al., 1998; Strompen et al., 1998; Jakoby et al., 2002). Factors such as NIM-INTERACTING2 (NIMIN-2), SNI1, NPR3, and NPR4 seem to be negative regulators which fine-tune NPR1-dependent gene expression (Li et al., 1999; Weigel et al., 2001; Chern et al., 2005a; Zhang et al., 2006; Zwicker et al., 2007).

The nuclear localization of NPR1 is essential for the expression of *PR* genes (Despres et al., 2000; Kinkema et al., 2000; Mou et al., 2003). Preceding cellular oxidative stress NPR1 is primarily found within the cytoplasm in an oligomeric form, though some notable exceptions exist (Kinkema et al., 2000; Mou et al., 2003; Le Henanff et al., 2009; Zhang et al., 2010c; Maier et al., 2011; Peraza-Echeverria et al., 2012; Shao et al., 2013). The production of SA and subsequent oxidative stress decreases cellular reduction potential enforcing an increased production of reducing agents (Mou et al., 2003). Thioredoxins, in particular thioredoxin H-type 3 (TRX-h3) and thioredoxin H-type 5 (TRX-h5), lead to the reduction of Cys156 and disassembly of the NPR1 oligomer (Mou et al., 2003; Tada et al., 2008). Monomeric NPR1 is then translocated to the nucleus via a bipartite nuclear localization signal (NLS) where it induces the expression of *PR1* (Kinkema et al., 2000; Maier et al., 2011). Consequently, inhibiting the formation or nuclear localization of NPR1 monomers decreases *PR1* expression while constitutive monomerization, as in the case of C82A and C216A point mutants, leads to increased *PR1* gene expression (Kinkema et al., 2000; Mou et al., 2003; Tada et al., 2008). Hence NPR1 is required as a monomer within the nucleus to induce SAR-related defense genes.

Remarkably NPR1 can be constitutively localized within the nucleus of tobacco and grapevine (Le Henanff et al., 2009; Maier et al., 2011). In spite of this, NPR1 within these species still seems to be dependent on SA (Le Henanff et al., 2009; Maier et al., 2011). Moreover, C82A and C216A mutants display even higher expression of *PR* genes following SAR induction (Mou et al., 2003). Confirmation of the SA-dependent nature of NPR1 came from Kinkema et al. (2000), proving that NPR1 nuclear localization was insufficient at inducing SAR in the absence of inducers such as INA (2,6-dichloroisonicotinic acid) or SA. Hence, mechanisms other than simple nuclear localization must play a role in controlling NPR1-dependent transcriptional processes.

## TGA Transcription Factors and NPR1

Although the N-terminal half of AtNPR1 exhibits low levels of transcriptional activity it is not likely to induce expression of *PR1* sufficiently (Zhang et al., 1999; Rochon et al., 2006). However, tobacco NPR1 harbors a stronger transactivation domain which is sensitive to SA (Maier et al., 2011). In yeast-1-hybrid screens, a section of NtNPR1 (1–315) exhibits transcriptional activity superior to the VP16 viral transactivation domain (Maier et al., 2011). Yeast cells expressing the Gal4 BD:NtNPR1 fusion protein in SA containing medium had much higher reporter gene activities than cells in media lacking SA (Maier et al., 2011). Hence the extent and way NPR1 is regulated differs between species reflecting their individual evolutionary histories and environments.

Expression of *PR1* in tobacco is highly dependent on *as-1-like* promoter elements known to be responsive to SA (Strompen et al., 1998). Several members of the TGA family of basic leucine zipper protein (bZIP) transcription factors associate with the *as-1-like* promoter element (Strompen et al., 1998; Zhang et al., 1999). Interestingly NPR1 has the ability to strongly interact with several of these transcription factors, namely; TGA1, TGA2, TGA3, TGA4, TGA5, TGA6, and TGA7 (Despres et al., 2000; Zhou et al., 2000; Johnson et al., 2003; Rochon et al., 2006). Interaction occurs predominantly within the nucleus where NPR1 activates TGA transcription factors by increasing their DNA binding affinity, evident through improved TGA2-*as-1-like* complex formation in the presence of wild-type NPR1 (Despres et al., 2000; Subramaniam et al., 2001; Fan and Dong, 2002). Two *as-1-like cis* elements can be found within the *PR1* promoter, a positive regulating element, *LS7*, and a negative regulating element, *LS5* (Lebel et al., 1998). Certain TGA transcription factors are able to associate with either of these elements, an association which is significantly enhanced by the presence of NPR1 (Despres et al., 2000). This suggests that NPR1 may not only serve to activate gene transcription but also suppress it in order to establish SAR (Despres et al., 2000). Further corroboration for the fundamental role of TGA transcription factors in regulation of defense gene expression was found in the promoters for SA-induced genes which showed an overrepresentation of the TGA2 binding sequence TGACTT (Ding et al., 2018).

In rice, *Arabidopsis* NPR1 binds to several bZIP transcription factors: rTGA2.1, rTGA2.2, rTGA2.3, rLG2 (Chern et al., 2001). Correspondingly, rTGA2.1 associates with the *Arabidopsis as-1-like* promoter element as well as the rice *RCH10* proximal promoter element (Chern et al., 2001). Tobacco contains several TGA transcription factors which are also capable of interacting with *Arabidopsis* NPR1, TGA2.1 and TGA2.2 (Niggeweg et al., 2000b). In addition both of these transcription factors are capable of binding to *as-1-like* elements (Niggeweg et al., 2000a,b). Tomato NPR1 was also found to associate with bZIP transcription factors which show high sequence similarity to the *Arabidopsis* TGA family of bZIP transcription factors (Zhang et al., 1999). Thus, a collectively conserved evolutionary role for NPR1 and TGA transcription factors is believed to exist in most if not all plant species.

Several studies have tried to address the *in vivo* role of TGA transcription factors (Pontier et al., 2001; Fan and Dong, 2002; Johnson et al., 2003; Zhang et al., 2003). In tobacco a TGA2 dominant-negative mutant resulted in increased *PR1*, *PR2* and *PR3* induction after SA treatment and enhanced disease resistance (Pontier et al., 2001). Contrastingly, a different dominant-negative TGA2 mutant led to decreased *PR* gene induction and enhanced disease susceptibility in both tobacco and *Arabidopsis* (Niggeweg et al., 2000b; Fan and Dong, 2002). These seemingly contrasting results are most likely due to the unknown interactions of dominant-negative mutants with other TGA transcription factors (Zhang et al., 2003). It seems that TGA2, TGA5, and TGA6 serve redundant roles in NPR1-dependent gene expression (Zhang et al., 2003). Either transcription factor is able to restore wild-type *PR* gene induction or basal expression levels in the *tga6-1tga2-1tga5-1* triple mutant (Zhang et al., 2003).

While *tga6-1tga2-1tga5-1* triple knockout mutants had reduced *PR* gene induction, basal levels of these genes were up to 50-fold higher (Zhang et al., 2003). This would suggest a negative role of TGA factors in basal *PR* expression yet a positive requirement for induction following SA perception. Yet, another study provides evidence that TGA2 is unable to bind to the *PR1* promoter in the absence of SA (Johnson et al., 2003). However, an elegant study by Rochon et al. (2006) clarified the conflicting evidence, showing that TGA2 and NPR1 are able to associate with the *PR1* promoter independently of each other in the absence of SA. Interestingly, NPR1 is capable of associating with TGA2 after SA treatment leading to *PR1* expression. The authors suggest that while TGA2 is a transcriptional repressor, NPR1 becomes a TGA2 transcriptional co-activator after perception of SA (Rochon et al., 2006). Indeed, Boyle et al. (2009) demonstrate that the N-terminal region of TGA2 is a non-autonomous repression domain required for association with *PR1 cis*-elements.

Interaction between TGAs and NPR1 is dependent on a functional ankyrin repeat domain within NPR1 (Zhang et al., 1999; Zhou et al., 2000; Despres et al., 2003). Although not essential to the interaction, the N-terminal domain of NPR1 also appears to be responsible for strengthening the interaction between NPR1 and certain TGAs (Zhou et al., 2000). Several *npr1* mutants deficient in mounting an effective SAR response are unable to interact with *Arabidopsis* TGA2, TGA3 as well as rice TGAs (Zhang et al., 1999; Despres et al., 2000; Zhou et al., 2000; Chern et al., 2001). These mutants, specifically *npr1-1, npr1-2, nim1-2* and *npr1-5*, have point mutations in the ankyrin repeat domain (Cao et al., 1994, 1997; Delaney et al., 1995; Glazebrook et al., 1996; Ryals et al., 1997; Shah et al., 1997).

Redox seems to play yet another essential role in the activity of both TGA transcription factors and NPR1. The C-terminal section of interacting TGA transcription factors is required for NPR1-TGA formation *in vitro* and *in vivo* (Zhang et al., 1999; Zhou et al., 2000; Fan and Dong, 2002). While TGA1 and TGA4 were initially considered unable to interact with NPR1, Despres et al. (2003) determined that following SA treatment these transcription factors were able to interact with NPR1 *in planta*. Specifically, residues unique to TGA1 and TGA4, Cys260 and Cys266, mediate the interaction (Despres et al., 2003). During non-induced conditions these residues form an intramolecular disulphide bridge which prevents TGA1 from interacting with NPR1, yet after SA treatment Cys260 and Cys266 are reduced and TGA1-NPR1 interaction occurs (Despres et al., 2003). Similarly, exchanging Cys260 and Cys266 for Asn and Ser, respectively, allows constitutive interaction with NPR1 in the absence of SA (Despres et al., 2003).

## NIM Interacting Proteins

An additional group of NPR1-interacting proteins are NIMINs (NIM INTERACTING) proteins (Weigel et al., 2005; Maier et al., 2011). These proteins are induced by SA or its functional analogs followed by nuclear localization where they interact directly with NPR1 forming a ternary complex with TGA factors (Weigel et al., 2001; Glocova et al., 2005; Weigel et al., 2005). In *Arabidopsis*, *35S:NIMIN1* overexpression abolishes the establishment of SAR and reduced *PR* expression (Weigel et al., 2005). However, overexpression of *NIMIN1-*2, which encodes for a mutant protein unable to bind to NPR1, results in near wild-type SAR induction and *PR* expression (Weigel et al., 2005). Moreover, knockout *nimin1-1* mutants showed increased *PR* expression after SA induction (Weigel et al., 2005). Similar results were obtained through overexpression of rice *NRR*, an ortholog of *NIMIN-2* and tobacco *NIMIN-2a* (Chern et al., 2005a). In addition, application of SA/INA to tobacco or *Arabidopsis* substantially reduces the NPR1 interaction potential of NIMIN proteins, specifically NIMIN-1/2 in *Arabidopsis* and NIMIN-2a/2b/2c in tobacco (Maier et al., 2011).

The aforementioned interaction is likely to be affected due to a conformational change which obscures the NIMIN binding motif in the C-terminal end of NPR1 (Maier et al., 2011). A single amino acid change in the C-terminal of the *nim1-4* mutant (Arg432Lys) severely impairs its potential to establish SAR (Ryals et al., 1997). Maier et al. (2011) concluded that this mutation rendered the interaction between NIMIN-1 and NIMIN-2 to NIM1-4 non-responsive to SA in both *Arabidopsis* and tobacco. Therefore, NIMIN is clearly involved in regulating *PR* expression through modulating NPR1 activity in response to SA.

In *Arabidopsis* NIMIN-1, NIMIN-2, and NIMIN-3 prevent each other from binding to NPR1, this interaction is dependent on the concentration of each protein and supports a theory whereby NIMIN proteins differentially interact with NPR1 (Hermann et al., 2013). In unchallenged *Arabidopsis* plants NIMIN-3 binds to NPR1 to prevent expression of *PR* genes (Hermann et al., 2013). Upon SA detection NIMIN-2 is quickly induced and although it is not involved in suppressing *PR* gene expression it seems to play an unknown role in immediate/early SA responses (Hermann et al., 2013). NIMIN-1 on the other hand delays the expression of *PR* genes, preventing premature activation (Hermann et al., 2013). Similarly, in challenged tobacco plants overexpression of NIMIN-2a does not prevent *PR* expression but rather delays its expression (Zwicker et al., 2007). Correspondingly, down-regulation of NIMIN-2a leads to earlier *PR* gene expression (Zwicker et al., 2007). Additionally these data suggest that NIMIN-2a may be involved in priming tissue distal from the primary site of infection, allowing a quicker response during secondary infection (Zwicker et al., 2007).

## Structure and Function of NPR1

Two domains are essential for the co-activator function of NPR1, the BTB/POZ domain in the N-terminal region as well as a cryptic transactivation domain in the C-terminal region (Rochon et al., 2006; Boyle et al., 2009). Exchanging the α2 and α3 helix residues, which constitute the core of the BTB/POZ domain, with Ala (A-Sub) or removing the first 110 amino acids (Δ110NPR1) of the domain abolishes *PR1* expression (Rochon et al., 2006). However, these mutations do not substantially reduce TGA2-NPR1 binding, providing evidence that the BTB/POZ domain is responsible for co-activation of TGA2 (Rochon et al., 2006). Boyle et al. (2009) were able to confirm this observation by restoring the inducible nature of *PR1* in *Arabidopsis* lines containing A-Sub or Δ110NPR1 in which a truncated TGA2, which lacks the N-terminal repression domain Δ43:TGA2, was coexpressed. The authors were able to demonstrate that the BTB/POZ domain physically interacts with the N-terminal repression domain of TGA2, negating its effect (Boyle et al., 2009).

Furthermore, transcriptional activation via the TGA2-NPR1 complex after treatment with SA requires the C-terminal transactivation domain and two essential cysteine residues, Cys521 and Cys529, in an oxidized state within this domain (Rochon et al., 2006). Surprisingly, while full length NPR1 tethered to a Gal4 DNA-binding domain lacks the ability to activate transcription in the absence of SA, the truncated Δ513:NPR1 C-terminal region containing the transactivation domain can (Rochon et al., 2006). A subsequent study showed that the N-terminal BTB/POZ domain inhibits the C-terminal transactivation domain in SA naïve cells through physical interaction, yet binding of SA to NPR1 disrupts this interaction through a conformational change (Wu et al., 2012b).

It was not until recently that two independent studies provided evidence that NPR1 and its paralogs directly interact with SA, providing invaluable insight into our understanding of SA perception (Fu et al., 2012; Wu et al., 2012b). In the first study, using conventional non-equilibrium ligand binding assays, NPR3 and NPR4 were shown to bind to SA with low and high affinity, respectively (Fu et al., 2012). Subsequently, a different approach utilizing equilibrium dialysis found that NPR1 too can bind to SA with an affinity similar to that of other known hormone-receptor interactions (Wu et al., 2012b). This interaction has been confirmed using three additional methods of detection, irrefutably setting the role of NPR1 as a *bone fide* SA receptor (Manohar et al., 2015). Binding to SA specifically requires Cys521 and Cys529 and the presence of a transition metal, preferably copper, to facilitate it (Wu et al., 2012b). Although orthologs of *Arabidopsis* NPR1 don’t harbor the same cysteine residues, the presence of similar residues with electronegative side-chains at comparable positions suggest some likelihood of parallel transition metal associations in other plant species (Wu et al., 2012b). This interaction enforces a conformational change in the C-terminal transactivation domain which reduces its affinity for the N-terminal BTB/POZ domain (Wu et al., 2012b). The authors further demonstrated that reducing conditions alone are not enough for disassembly of the NPR1 oligomer and suggested that the SA-induced conformational change was required for full disassembly (Wu et al., 2012b). Thus, the function of NPR1 is enforced through a conformational changes which rely on direct interaction with SA.

## Paralogs of NPR1

Paralogs of NPR1, namely NPR3 and NPR4, appear to be negative regulators of *PR* expression (Zhang et al., 2006). Similar structure in these proteins seems to extend to functional similarities, from perception of SA to binding of TGAs (Despres et al., 2000; Kinkema et al., 2000; Subramaniam et al., 2001; Fan and Dong, 2002; Mou et al., 2003; Rochon et al., 2006; Zhang et al., 2006; Shi et al., 2013). Initial research suggested that NPR4 could be a positive regulator of disease resistance as *PR* expression priming was compromised in *npr4-2* mutants (Liu et al., 2005). However, *npr3 npr4* double mutants displayed increased *PR* gene expression and increased disease resistance indicating that *npr4-2* contributes to increased *PR* expression in *npr3 npr4* double mutants (Zhang et al., 2006). The redundancy of these proteins was also demonstrated through complementation with NPR3 or NPR4 (Zhang et al., 2006). Interestingly, NPR3 and NPR4 have also been shown to increase JA-dependent gene transcription and *de novo* JA synthesis following the accumulation of SA likely by promoting the degradation of JA repressing JAZ (JASMONATE ZIM DOMAIN) proteins (Liu et al., 2016). This suggests that NPR3 and NPR4 are essential in preventing disease caused by necrotrophic pathogens on tissues affected by ETI-triggered PCD (Liu et al., 2016). Several studies stimulated substantial debate regarding the exact role of NPR3/NPR4 in defense responses (Fu et al., 2012; Wu et al., 2012b; Kuai et al., 2015). However, their role as co-repressors of SA-inducible defense gene expression is clearly demonstrated by Ding et al. (2018).

Originally, NPR3 and NPR4 were proposed to primarily function as E3 ligases in a model by Fu et al. (2012). The authors demonstrated that NPR3 and NPR4 possess differing affinities for SA, thus allowing them to effectively regulate NPR1-dependent gene expression through CUL3-mediated proteasome degradation of NPR1 (Fu et al., 2012). In naïve cells with low SA concentrations, NPR4 which possess the highest affinity binds to NPR1 preventing ill-timed *PR* expression (Fu et al., 2012). Else, in SAR induced cells higher SA concentrations prevent NPR4-NPR1 association whereas NPR3 gains the ability to interact with NPR1, preventing NPR1-mediated suppression of the HR (Rate and Greenberg, 2001; Fu et al., 2012). Thus, NPR1 turnover rate and ultimately the induction of SAR is determined by the concentration gradient of SA from the initial site of pathogen infiltration to distal tissues. However, the model proposed by Fu et al. (2012) is inconsistent with the ostensible genetic redundancies between *NPR3* and *NPR4* (Kuai et al., 2015). Thus suggesting that NPR3 and NPR4 rather serve redundant roles, in contrast to independently functioning as SA receptors (Kuai et al., 2015). Additionally, no observable interaction occurs between NPR1 and NPR3/NPR4 in yeast-2-hybrid assays or NPR3/NPR4 and Cul3A in co-immunoprecipitation assays (Ding et al., 2018). This suggests, at least, that determining whether NPR3 and NPR4 participate in post-translational modification of NPR1 requires further study.

Instead, Ding et al. (2018) demonstrated that NPR3 and NPR4 are transcriptional co-repressors of SA-induced defense gene expression which function in parallel and independently of NPR1. The authors identified *npr4-4D* (Arg419Gln), a gain-of-function mutation which renders the mutant protein insensitive to SA and constitutively represses SA-inducible defense genes (Ding et al., 2018). Interestingly, mutation of the equivalent amino acid in NPR1 renders it insensitive to SA and is arguably the reason why NIM1-4 does not dissociate from NIMIN1 and NIMIN2 in the presence of SA (Ryals et al., 1997; Maier et al., 2011). An equivalent mutation introduced into NPR3 (Arg428Gln) similarly enables it to suppress defense signaling in the presence of SA, confirming the redundant roles of NPR3/NPR4 (Ding et al., 2018). Transcriptional repression of SA-inducible defense genes was shown to rely on a conserved motif (VDLNETP) within the C-terminal domain of NPR3 and NPR4 with similarity to the ethylene responsive element binding factor associated amphipathic repression motif (EAR; L/FDLNL/F(x)P) (Ohta et al., 2001; Ding et al., 2018). Furthermore, NPR3/NPR4 work together with TGA2/TGA5/TGA6 to suppress the expression of SA-inducible defense genes in SA naïve cells (Ding et al., 2018). The authors also confirmed that NPR3 and NPR4 bind to SA with high affinity. Meanwhile NPR4-4D, while still able to bind to TGA2 and form homodimers, showed a significantly lower (250-fold) binding affinity, demonstrating that R419 is essential in the binding of SA (Ding et al., 2018). Similarly, the NPR1^R432Q^ mutant displays significantly lower SA binding affinity compared to wild-type NPR1 with no apparent effects on interactions with TGA2 or NIMIN1 in yeast-2-hybrid assays (Ding et al., 2018). Thus, both NPR3 and NPR4 are *bone fide* SA receptors with highly similar functionality to that of NPR1, albeit in opposition regarding SA-inducible defense gene expression.

The *BLADE-ON-PETIOLE 1* (*BOP1*) and *BOP2* genes encode proteins with structure similar to other NPR1-like proteins, containing both N-terminal BTB/POZ and C-terminal ankyrin repeat domains (Ha et al., 2003; Ha et al., 2004). However, the C-terminal of several BOP-like proteins in several plant species lack essential features characteristic of defense-related NPR1-like proteins such as a clear bipartite NLS, NIMIN1/2 binding region and the highly conserved NPR1 Arg432 residue (Backer et al., 2015). These differences seemingly translate into functional variation as *bop1 bop2* double mutants display an unaltered wild-type response to *Pseudomonas* infection as well as SA application (Hepworth et al., 2005; Canet et al., 2010a). However, some evidence of defense-related functions exist with BOP1 and BOP2 being implicated in the resistance-inducing activity of MeJA (Canet et al., 2012). Nonetheless, BOP1 and BOP2 are considered transcriptional co-activators which function redundantly and share similar transcriptional patterns, being expressed primarily at the base of lateral organs (Ha et al., 2004; Hepworth et al., 2005; Norberg et al., 2005; Ha et al., 2007; McKim et al., 2008; Jun et al., 2010). Correspondingly, the overwhelming majority of evidence indicates that BOPs are vital to the growth and development of lateral organs (Ha et al., 2004, 2007; Hepworth et al., 2005; Norberg et al., 2005; McKim et al., 2008). Even though they lack a defined NLS, BOPs can be found within the cytoplasm and nucleus of *Arabidopsis* (Ha et al., 2004; Hepworth et al., 2005). Thus unsurprisingly, BOPs influence transcriptional processes through interaction with TGA transcription factors (Hepworth et al., 2005; Wu et al., 2012a). Of relevance is PERIANTHIA (PAN), a TGA transcription factor with known significance to developmental processes in *Arabidopsis* (Chuang et al., 1999; Hepworth et al., 2005). Interestingly, BOPs have also been implicated in the lignin biosynthesis pathway (Khan et al., 2012). Hence, members of the NPR1-like family together make up one of the most important groups of proteins to study in the field of molecular biology of plant health and development.

## WRKY Transcription Factors and NPR1

Microarray of the *Arabidopsis* transcriptome during SAR revealed that not all genes co-regulated with *PR1* contain the expected TGA binding site in their promoters (Maleck et al., 2000). Instead, W-box *cis*-elements which specifically bind WRKY transcription factors are more common suggesting that WRKY transcription factors might repress a subset of SA-inducible genes, which is alleviated during SAR (Maleck et al., 2000). Indeed, *wrky38* and *wry62* single mutants and to a greater extent *wrky38wrky62* double mutants display enhanced disease resistance and *PR1* expression while overexpression has the opposite outcome (Kim et al., 2008). However, many WRKY transcription factors are positively associated with defense signaling, thus the role of this family in defense is complex (Wang et al., 2006; Zheng et al., 2006; Lai et al., 2008). These transcription factors are involved in defense against a wide range of pathogens, with 43 out of 74 WRKY transcription factors in *Arabidopsis* being linked to pathogenic stress and response to SA (Dong et al., 2003; Ulker and Somssich, 2004; Pandey and Somssich, 2009).

Accordingly, W-box *cis*-elements are found in several indispensable SA-inducible defense response gene promoters including that of *ICS1, TL1-binding transcription factor (TBF1)* and *PR1* (Eulgem et al., 2000; Wildermuth et al., 2001; Turck et al., 2004; Pajerowska-Mukhtar et al., 2012). Furthermore, the presence of multiple W-boxes within the *NPR1* promoter suggest that *NPR1* may be transcriptionally regulated in this manner (Yu et al., 2001). Yu et al. (2001) demonstrated that WRKYs are likely involved in positively regulating the expression of *NPR1*, although the exact WRKY is yet to be identified. Nonetheless, WRKY transcription factors are regulated in both NPR1-dependant and independent manners (Yu et al., 2001; Dong et al., 2003; Wang et al., 2006; Mao et al., 2007; Spoel et al., 2009).

Interestingly, the *CmYLCV* promoter from *Cestrum yellow leaf curling virus* contains both the *as-1* and W-box *cis*-elements in close proximity which associate with both TGA3 and WRKY53 (Sarkar et al., 2018). These elements are essential to the SA-inducibility of the promoter, suggesting that in certain instances both TGA and WRKY transcription factors may work together to regulate transcription (Sarkar et al., 2018). Surprisingly, not only do these transcription factors interact, they require functional NPR1 to induce expression of *Gus* under control of the *CmYLCV* promoter after treatment with SA (Sarkar et al., 2018). Given the complexity of the interactions described here the possibility exists that NPR1 is both positively and negatively regulated by various WRKY transcription factors, although this requires further investigation.

## ER Resident Proteins and NPR1

Several genes involved in the secretory pathway are upregulated by NPR1, the most notable are *LUMINAL BINDING PROTEIN 2* (*BiP2*), *Sec61*α, *DEFENDER AGAINST APOPTOTIC DEATH 1* (*DAD1*), and *CALRETICULIN* 3 (*CRT3*) (Wang et al., 2005; Pajerowska-Mukhtar et al., 2012). These secretion-related genes all have a common promoter *cis*-element (*TL1*) which is bound by TBF1 a heat shock factor-like transcription factor instrumental in the growth-defense transition (Wang et al., 2005; Pajerowska-Mukhtar et al., 2012). Increased expression of secretory pathway genes most likely accommodates increased PR protein production during SAR, ensuring proper protein folding (Wang et al., 2005). Support for this conclusion came from mutants of *BiP2*, *Sec61*α, and *DAD1* which all had reduced PR1 secretion after BTH treatment and compromised defense against *P. syringae* (Wang et al., 2005). Similarly, *tbf1* mutants have unaltered *PR1* transcript and protein levels yet significantly less protein is secreted into the apoplast (Pajerowska-Mukhtar et al., 2012). Furthermore, the link between TBF1 and NPR1 is evident from *tbf1* and *npr1-1* mutants which are both similarly compromised in the expression of *BiP2* and *CRT3* (Pajerowska-Mukhtar et al., 2012). Thus, the expression of *NPR1* and *TBF1* are likely co-dependent with the authors suggesting that TBF1 might control *NPR1* directly through *TL1* elements in the promoter, or indirectly through WRKYs, while NPR1 may control *TBF1* through TGAs directly or WRKYs indirectly as both contain the appropriate elements in their promoters, respectively.

## NPR1 in Priming

Priming is a process which enhances plant defense responses, enabling earlier and stronger induction of defense genes and enhanced pathogen resistance (Prime et al., 2006). In fact, SAR prepares a plant to defend against future pathogenic stress through priming (Conrath et al., 2002; Prime et al., 2006). Thus unsurprisingly, NPR1 is essential in SA-induced priming in *Arabidopsis* (Kohler et al., 2002; Jung et al., 2009). Of note are pathogen-responsive mitogen-activated protein kinase 3 (MPK3) and MPK6 which are essential to SAR and SA-mediated priming of defense responses (Beckers et al., 2009). Following application of BTH, MPK3/MPK6 mRNA and inactive unphosphorylated proteins accumulate (Beckers et al., 2009). Interestingly, the authors demonstrated that priming of MPK3/MPK6 is NPR1-dependant as *npr1* mutant *Arabidopsis* plants fail to display the same response. Yi and Kwon (2014) and Yi et al. (2015) demonstrated the importance of this finding as NPR1-dependant priming affects early signaling events, such as flg22-triggered MAPK activation.

Furthermore, priming has been shown to affect the progeny of primed *Arabidopsis* as descendants display enhanced resistance against biotic stresses without additional treatment (Luna et al., 2012; Rasmann et al., 2012; Slaughter et al., 2012). This transgenerational immune memory requires functional NPR1 (Luna et al., 2012). Transgenerational immune memory relies, at least in part, on increased H3K9 acetylation of the *PR1*, *WRKY6* and *WRKY53* promoters (Luna et al., 2012). Quite surprisingly, the histone deacetylase HDAC19 was shown to be both SA- and NPR1-dependant (Choi et al., 2012). Meanwhile, NPR1 is involved in BTH and *Psm* induced increases in H3K4 trimethylation and subsequent gene activation of the *WRKY6*, *WRKY29* and *WRKY53* promoters (Jaskiewicz et al., 2011). Together these studies suggest a role for NPR1 in histone modification to enforce priming of SA-induced defense genes, however, understanding the exact part that NPR1 plays in these processes requires further investigation.

## Post-Translational Modification of NPR1

An important topic which adds to the complexity of NPR1-dependant transcriptional regulation is that of post-translation modification. The importance of NPR1 post-translational modification is exemplified by regulation of the oligomer-monomer transition in which NPR1 Cys156 is *S*-nitrosylated by *S*-nitrosoglutathione (GSNO) promoting the existence of NPR1 in its oligomeric form, opposing the action of SA-induced thioredoxins (Tada et al., 2008).

Additionally, proteasome-mediated turnover of NPR1 within the nucleus is a requirement for the complete induction of SAR (Spoel et al., 2009). While the antagonistic effects of ABA and SA, which promote and protect against proteasome-mediated degradation, respectively, maintain homeostasis and ensure appropriate defense-related gene expression (Ding et al., 2016). Cullin 3 (CUL3) E3 ligase-facilitated ubiquitinylation and subsequent proteasome degradation is initiated within the N-terminal IκB-like phosphodegron motif of NPR1 (Spoel et al., 2009). Phosphorylation of Ser11/15 present in the phosphodegron motif signals proteasome-mediated degradation (Spoel et al., 2009). Yet, even though degradation of NPR1 is reduced and basal resistance is elevated in npr1^S11A/S15A^, high levels of accumulated NPR1 within the cell prevent HR and the establishment of SAR (Spoel et al., 2009). Compromised induction of SAR is also observed in mutants of NPR1-dependent genes *wrky18* and *wrky38 wrky62*, and similarly in *cul3a cul3b* mutants in which NPR1 is not degraded (Spoel et al., 2009). Hence, turnover seems to be necessary for effective activity of NPR1 (Spoel et al., 2009). This is somewhat expected as inherent instability of transcription factors necessitates turnover in order to preserve peak expression of target genes and is thus conceivably so for co-activators (Salghetti et al., 2000; Collins and Tansey, 2006; Spoel et al., 2009). However, NPR1 does not interact directly with CUL3 and E3 ligases, likely requiring substrate adapters to facilitate degradation, however, attempts to uncover such adapters have not been conclusive (Dieterle et al., 2005; Spoel et al., 2009; Fu et al., 2012).

Interestingly, small ubiquitin-like modifier 3 (SUMO3), which is positively involved in SA-induced defense gene expression, interacts with and sumoylates NPR1 following SA treatment (Wang et al., 2006; van den Burg et al., 2010; Saleh et al., 2015). This interaction requires a SUMO-interaction motif (VIL)-(VIL)-x-(VIL) found within the ankyrin repeat domain of NPR1 (Saleh et al., 2015). Sumoylation alters the association of NPR1 with WRKY and TGA transcription factors, decreasing and increasing association, respectively (Saleh et al., 2015). In addition to the IκB-like phosphodegron motif at Ser11/15, another exists at Ser55/59 and their phosphorylation status influences the ability of SUMO3 to sumoylate NPR1 (Saleh et al., 2015). Phospho-mimic npr1^S55D/S59D^ prevents NPR1 sumoylation while npr1^S11D/S15D^ enhances interaction with SUMO3 and leads to further sumoylation (Saleh et al., 2015). Additionally, SUMO3 is required for phosphorylation of Ser11/15 forming a signal amplification loop which activates more NPR1 increasing defense gene activation and simultaneously targeting NPR1 for ubiquitinylation and degradation by the 26S proteasome (Spoel et al., 2009; Saleh et al., 2015). Together with the results obtained by Spoel et al. (2009), this work emphasizes the importance of NPR1 stability, through post-translational modification, to fine-tune NPR1-dependant defense responses.

Thus unsurprisingly, several kinases have been implicated in the phosphorylation of NPR1 (Xie et al., 2010; Lee et al., 2015). A pathogen-responsive member of the sucrose non-fermenting 1 (SNF1)-related kinase 3 (SnRK3) subgroup, PROTEIN KINASE SOS2-LIKE5 (PKS5) physically interacts with NPR1 (Xie et al., 2010). The authors demonstrate that PKS5 phosphorylates the C-terminal region of NPR1, which contains the Cys-oxidized transactivation domain as well as the bipartite NLS. The *Arabidopsis pks5* mutant as is with the *npr1^S11A/S15A^* mutant displays reduced expression of *WRKY38* and *WRKY62* (Spoel et al., 2009; Xie et al., 2010). Thus it seems that through phosphorylation of NPR1, PKS5 positively regulates the expression of *WRKY38* and *WRKY62* (Xie et al., 2010).

Similarly, SNF-1 RELATED PROTEIN KINASE 2.8 (SnRK2.8) interacts with and phosphorylates NPR1, specifically Ser589 and likely also Thr373, which are required for nuclear import of NPR1 and subsequent *PR1* gene expression (Lee et al., 2015). Interestingly, SnRK2.8 is produced in response to SA-independent systemic signals and has been implicated in the induction of systemic immunity (Lee et al., 2015). It is possible that, similar to SnRK2.6, nitric oxide (NO) might play a role in SnRK2.8 activation as it plays a proven role in the import of NPR1 monomers into the nucleus of cells in distal tissues during SAR (Lindermayr et al., 2010; Lee et al., 2015). Furthermore, Ser589 resides within the second NLS found in NPR1 (NLS2) (Kinkema et al., 2000). The authors suggest a model by which SA-dependent NPR1 nuclear import, for which NLS1 is required, is predominant close to the site of infection, while distal tissues with only slightly elevated levels of SA, rely on phosphorylation of NLS2 by SnRK2.8 (Kinkema et al., 2000; Lee et al., 2015). Therefore, multisite phosphorylation is clearly a defining feature of NPR1 function and warrants further investigation.

## Circadian Rhythm and NPR1

In plants the circadian clock is crucial for synchronizing immune strategies, while redox signaling plays an important role in its implementation (Karapetyan and Dong, 2018). SA levels oscillate throughout the day in a circadian rhythm (Goodspeed et al., 2012). This oscillation is involved in establishing the redox rhythm and influencing the expression of circadian clock genes (Zhou et al., 2015). Captivatingly, the expression of *TIMING OF CAB2 EXPRESSION 1* (*TOC1*), an evening circadian clock gene, is upregulated by the application of SA. However, the timing of its expression does not change irrespective of whether SA is applied at dawn or dusk (Zhou et al., 2015). Due to the redox sensitivity of NPR1, Zhou et al. (2015) hypothesized that NPR1 might play a role in the expression of *TOC1*. Indeed, they demonstrated that basal and SA-induced expression of *TOC1* was reduced and abolished, respectively, in *npr1* mutants (Zhou et al., 2015). This concept was further supported as NPR1 displayed increased association with TGA-binding sites of the *TOC1* promoter in a SA-dependent manner (Zhou et al., 2015). The NPR1 monomer also shows a circadian oscillation, peaking at night (Zhou et al., 2015). The influence that NPR1 has on *TOC1* is reliant on its translocation into the nucleus as *trx-h3 trx-h5* mutants showed decreased basal *TOC1* expression and decreased responsiveness to SA (Zhou et al., 2015). Thus, oscillation in SA and subsequent redox changes drive the nuclear translocation of NPR1, which is required for the regulation of *TOC1* (Zhou et al., 2015). However, these findings do not fully explain why SA-application at dawn showed a delayed induction of *TOC1* until dusk. Through mathematical modeling and *in planta* confirmation it was shown that NPR1 regulates the morning clock gene *LATE ELONGATED HYPOCOTYL* (*LHY*), a known antagonist of *TOC1* (Zhou et al., 2015). This study underpins the importance of NPR1 in defense responses as well as the circadian clock, by interlacing these processes the plant can prioritize growth over increased immunity at night while increasing immunity at dawn when the threat from pathogens is highest (Nozue et al., 2007; Bhardwaj et al., 2011; Korneli et al., 2014; Zhou et al., 2015).

## NPR1 in Transgenic Crops

*Arabidopsis NPR1* has been overexpressed in a multitude of agricultural crops and can enhance resistance to a variety of biotrophic and necrotrophic pathogens (Cao et al., 1998; Chern et al., 2001, 2005b; Lin et al., 2004; Makandar et al., 2006; Xujing et al., 2006; Malnoy et al., 2007; Potlakayala et al., 2007; Yuan et al., 2007; Wally et al., 2009; Parkhi et al., 2010b; Le Henanff et al., 2011; Kumar et al., 2013; Dutt et al., 2015; Molla et al., 2016). This indicates that a high level of functional conservation likely exists in all plant species. However, overexpression studies in rice and strawberry also demonstrated the negative influences constitutive overexpression of *AtNPR1* can have on certain crops (Fitzgerald et al., 2004; Quilis et al., 2008; Silva et al., 2015). Though, specifically expressing *AtNPR1* in only the green tissues of rice, using the P_D54O-544_ promoter, conferred resistance to sheath blight disease caused by the fungus *Rhizoctonia solani* without any detrimental phenotypic effects (Molla et al., 2016). This would suggest that more targeted expression of *AtNPR1* might benefit strawberry as well as any other crops which exhibit sensitivity to global overexpression.

Nevertheless, *AtNPR1* overexpression in crops such as wheat, tomato, carrot, soybean, canola, citrus, tobacco, and cotton display significantly improved disease resistance and even crop yield without any negative phenotypic effects (Lin et al., 2004; Makandar et al., 2006; Potlakayala et al., 2007; Boller and He, 2009; Wally et al., 2009; Parkhi et al., 2010a,b; Gao et al., 2013; Kumar et al., 2013; Matthews et al., 2014). Interestingly overexpression of *AtNPR1* seems to have a negligible effect on basal defense gene expression in many crops, while significantly increasing the response time and strength of defense responses (Wally et al., 2009; Zhang et al., 2010b; Kumar et al., 2013; Boscariol-Camargo et al., 2016). Remarkably, tobacco overexpressing *AtNPR1* also displayed increased resistance to the herbivore *Spodoptera litura* (Meur et al., 2008). Thus, in many cases increasing NPR1 expression not only increases broad spectrum disease resistance but does so without negative impacts on plant growth.

Overexpression of Rice *NPR1* (*OsNPR1/NH1*) in *Arabidopsis* is able to complement the *npr1* mutant, however, several negative consequences are observed, including enhanced herbivore susceptibility (Yuan et al., 2007). This, however, suggests a role for NH1, like that of AtNPR1, in crosstalk between the SA- and JA-defense signaling pathways. Yet, these observations together with the negative phenotypic effects observed during overexpression of *AtNPR1* in rice while such deleterious effects are absent in many other crops, would suggest that some notable differences exist between species regarding the regulation of NPR1. The high basal level of SA in rice which remains unaltered following pathogen infection contrasts with that of *Arabidopsis* and tobacco and supports such a theory (Quilis et al., 2008; Dempsey et al., 2010). Therefore, although NPR1 may serve functionally conserved roles in all plant species, the underlying mechanisms which regulate NPR1-dependant pathways need to be understood for the species under investigation.

Overexpression of *AtNPR1* orthologs from several plant species have also been studied (Malnoy et al., 2007; Potlakayala et al., 2007; Le Henanff et al., 2009, 2011; Shi et al., 2010; Chen et al., 2012b; Yocgo et al., 2012; Zhang et al., 2013; Wang et al., 2017). Apple *AtNPR1* orthologs *MhNPR1* (*Malus hupehensis*) and *MpNPR1* (*Malus pumila*) enhanced resistance to several important pathogens in *Malus x domestica* (Malnoy et al., 2007; Chen et al., 2012b). Likewise, overexpression of *LhSorNPR1* from the oriental hybrid lily ‘Sorbonne’ in *Arabidopsis* increased wild-type resistance to *P. syringae* (Wang et al., 2017). In tobacco, overexpression of *MhNPR1* increased resistance to *B. cinerea* and interestingly, salt tolerance (Zhang et al., 2012, 2014). Similarly, complementation of *Arabidopsis npr1* using *VaNPR1.1* (*Vitis aestivalis* cv. Norton) increased salt tolerance (Zhang et al., 2013). Additionally, *Arabidopsis npr1* mutants are complemented using *BnNPR1* (*Brassica napus*) while overexpression in *B. napus* enhanced resistance to *P. syringae* without any obvious negative effects, emphasizing similarity between NPR1-dependant defense responses in these two species (Potlakayala et al., 2007). However, *NPR1* incompatibility between species is made apparent as apical dominance is affected in *Arabidopsis* overexpressing *VvNPR1.1* (Le Henanff et al., 2011). These results support the highly versatile and important role of NPR1 in studied plant species. For a thorough review on its potential uses in transgenic crop protection, see Silva et al. (2018).

## Conclusion and Future Directions

**FIGURE 2:**
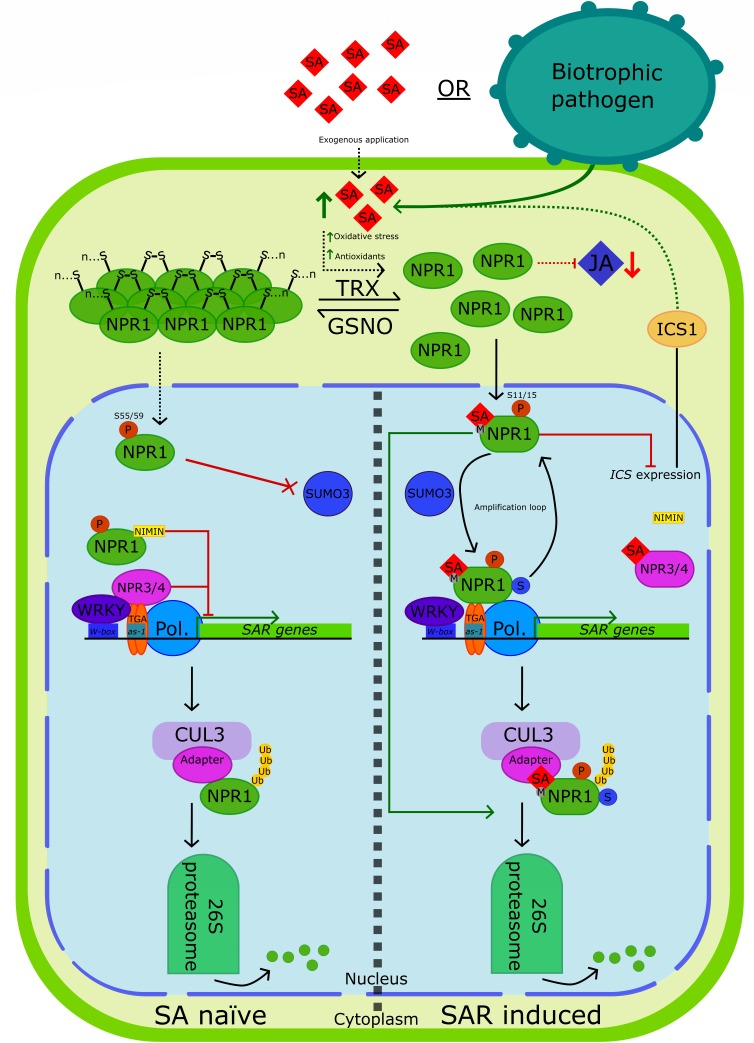
A working model of NPR1, NPR3, and NPR4. The left side of the diagram, partially separated by a dashed line, depicts the regulation of NPR1-dependant defense genes in SA naïve cells. At low SA concentrations, *S*-nitrosylation of NPR1^Cys156^ by GSNO encourages the existence of NPR1 in its oligomeric form. The oligomeric form of NPR1 is predominantly found within the cytoplasm. To prevent the uninduced expression of SAR-related genes, several mechanisms are in place to suppress NPR1-dependant defense gene expression. Phosphorylation of NPR1^Ser55/59^ suppresses defense gene expression and prevents sumoylation of NPR1 by SUMO3, an important aspect of NPR1 activation. The N-terminal BTB/POZ domain of NPR1 interacts with and suppresses the function of the C-terminal transactivation domain of NPR1. Additionally, NIMIN proteins interact with NPR1 to suppress gene expression. Paralogs of NPR1, NPR3, and NPR4, interact with TGA2/TGA5/TGA6 to further suppress transcription. Certain WRKY transcription factors act as transcriptional repressors of a subset of SAR-related genes. Finally, NPR1 is degraded by the 26S proteasome following CUL3-mediated ubiquitinylation. However, NPR1 is unable to directly interact with CUL3 and E3 ligases, likely requiring a substrate adapter. The right side of the diagram depicts NPR1 regulation in SAR-induced cells where SA concentration is elevated either due to exogenous application of SA/one of its functional analogs or during biotrophic/hemibiotrophic pathogen challenge. Increased oxidative stress and subsequent increases in antioxidant production leads to the reduction of NPR1^Cys156^, specifically by thioredoxins, leading to the disassembly of the NPR1 oligomer. Within the cytoplasm, NPR1 antagonizes the JA-defense response pathway. Monomeric NPR1 is then translocated to the nucleus via the action of a bipartite nuclear localization signal. Within the nucleus, NPR1 suppresses the expression of *ICS1* which is essential to SA synthesis in response to pathogenic stress, forming a negative feedback loop. Phosphorylation of NPR1^Ser11/15^ within the N-terminal IκB-like phosphodegron motif both enhances interaction with SUMO3 and targets NPR1 for ubiquitinylation and degradation by the 26S proteasome. Sumoylation of NPR1 by SUMO3 also increases and decreases association of NPR1 with TGA and WRKY transcription factors, respectively. SUMO3 is also required for phosphorylation of NPR1^Ser11/15^, creating an amplification loop which leads to the activation of more NPR1, increasing SAR-related gene expression. Interaction of SA and NPR1 requires a transition metal. Following binding of SA to NPR1 a conformational change of C-terminal transactivation domain of NPR1 decreases its affinity for the inhibitory N-terminal BTB/POZ domain. In turn, the BTB/POZ domain of NPR1 interacts with the N-terminal repression domain of TGA transcription factors, thereby activating transcription. Furthermore, binding of SA to NPR1 alters its interaction with NIMINs, relieving repression. Moreover, binding of SA to NPR3/NPR4 diminishes their ability to suppress SAR-related gene expression. Turnover of NPR1 through degradation by the 26S proteasome is essential to preserving peak gene expression and is required for the complete induction of SAR.

Since its discovery more than 20 years ago, NPR1 has been the focus of countless studies. During this time several NPR1-dependant pathways have been uncovered as have many of the complex mechanisms governing the regulation of NPR1 (Figure 2). However, much is still left unanswered, owed to the multifaceted relationships that exist between NPR1, its paralogs and their interacting partners. Truly grasping the extent of such interactions requires an increased effort to discover novel interactions in different species, tissues and during plant–pathogen specific interactions. A topic pursuant to this which is severely underrepresented in the literature is that of tissue specific regulation of NPR1-dependant pathways. Microarray data suggests that significant differences exist regarding the expression of *NPR1*-like genes across various tissues in *Arabidopsis* (Shi et al., 2013). Similarly, the expression of avocado *NPR1*-like genes exhibit unique spatial preferences (Backer et al., 2015). The importance of these observations are highlighted in *Arabidopsis npr3* knockout mutants which display increased resistance to *P. syringae* on developing flowers but not leaves (Shi et al., 2013).

Important aspects of the regulation of NPR1 function and homeostasis which require further attention are that of post-translational modification and proteasome degradation of NPR1. Important questions have been raised regarding these processes, including whether NPR3 and NPR4 act as E3 ligases which lead to the ubiquitinylation of NPR1. Surprisingly, even though Fu et al. (2012) suggested such a role, a more recent report failed to detect interactions between either NPR1 and NPR3/NPR4 or NPR3/NPR4 and CUL3A (Ding et al., 2018). Given the importance of NPR1 turnover to maintain optimal NPR1-dependant gene expression and the role post-translational modifications have in this process, it is imperative to further characterize and understand the process. Discovering how exactly NPR1 is ubiquitinylated by CUL3 given the absence of direct interaction could increase our understanding of post-translational modification as a means of regulating the function of NPR1.

Given that NPR1, NPR3, NPR4, BOP1, and BOP2 all function in the regulation of various transcriptional processes, it is hard to ignore the possibility that NPR2 may serve a similar yet undefined function. This is especially true since *NPR2* is induced by biotic stress and was shown to play a significant role in the perception of SA (Canet et al., 2010b). Phytohormones SA, JA and ET are known to promote leaf senescence and notably, various WRKY and bZIP transcription factors are involved (Zhao et al., 2016). Furthermore, an essential component of SA-induced leaf senescence in *Arabidopsis*, MAPK6, influences the activity and gene expression of *NPR1* (Chai et al., 2014). Therefore it is not surprising that *Arabidopsis npr1-5* null mutants which are impaired in SA biosynthesis suppress precocious leaf senescence characteristic of *pat14* mutants (Zhao et al., 2016). Together, these data clearly suggest that NPR1-dependant signaling is involved in senescence. Interestingly, Shi et al. (2013) noted that NPR2 transcripts were most abundant in senescent tissue, thus it is conceivable that NPR2 may serve a similar or even a more direct role in senescence. Conversely, NPR2 may simply be a redundant or non-functional paralog of NPR1. In either case, it would be worthwhile to investigate and determining which transcription factors interact with NPR2 may be a good place to start.

Given the complex nature of the NPR1-like protein family and uncertainty surrounding the aspects mentioned above, caution should be exercised regarding generalizing statements concerning functions *in planta* and across species. Instead emphasis should be placed on describing temporal, spatial and plant–pathogen interaction specific functions. Nonetheless, continued research on the NPR1-like protein family is warranted and will undoubtedly bring forth novel insights into the molecular pathways involved in plant stress responses and development.

## Author Contributions

RB conceptualized, drafted, and reviewed the manuscript. SN reviewed and assisted in drafting the manuscript. NvdB conceptualized, reviewed, and assisted in drafting the manuscript. All authors contributed to and approved the final manuscript.

## Conflict of Interest Statement

The authors declare that the research was conducted in the absence of any commercial or financial relationships that could be construed as a potential conflict of interest.
